# Cytokines, Serological, and Histopathological Assessment of Recombinant Vaccination Strategies for Combatting Infectious Bursal Disease in Broiler Chickens

**DOI:** 10.3390/vaccines12010027

**Published:** 2023-12-26

**Authors:** Mahmoud S. Gewaily, Fares El-Khyat, Abd Elnaby Tahoon, Mohammed Al-Rasheed, Safaa E. Abdo, Ahmed Gado, Mohamed Elmasry, Mahmoud M. Ismail

**Affiliations:** 1 Department of Anatomy and Embryology, Faculty of Veterinary Medicine, Kafrelsheikh University, Kafrelsheikh 33516, Egypt; 2 Department of Poultry and Rabbit Diseases, Faculty of Veterinary Medicine, Kafrelsheikh University, Kafrelsheikh 33516, Egyptmahmoud.ismail@vet.kfs.edu.eg (M.M.I.); 3 Animal Health Research Institute, Kafrelsheikh Branch, Kafrelsheikh 33511, Egypt; 4 Department Clinical Sciences, College of Veterinary Medicine, Avian Research Center, King Faisal University, P.O. Box 400, Al-Ahsa 31982, Saudi Arabia; malrasheed@kfu.edu.sa; 5 Department of Animal Wealth Development, Faculty of Veterinary Medicine, Kafrelsheikh University, Kafrelsheikh 33516, Egypt; 6 Department of Poultry and Fish Diseases, Faculty of Veterinary Medicine, Damanhour University, Damanhour 22511, Egypt; 7 Agricultural Research Center, Animal Production Research Institute, Animal Production Research Station, Sakha, Kafrelsheikh 33511, Egypt

**Keywords:** IBDV, recombinant vaccines, immune complex, histopathology, cytokines

## Abstract

Infectious bursal disease (IBD) represents a greatly transmissible viral disease found worldwide, causing significant health and production challenges in young chickens. The aim of this research was to assess the immune reaction induced by different vaccines targeting IBD. These vaccines included recombinant (Vac1; HVT-IBD vector), immune complex (Vac2; Bursa-Plex^®^), and intermediate plus (Vac3; Bursine plus) IBD vaccines. Our assessment relied on serological and histopathological analyses, as well as the pattern of immune-related cytokine expression in the bursal tissue. The vaccinated groups, along with a control positive (CP) group, were subjected to a vvIBDV challenge on their 28th day of life, while the control negative (CN) group received a mock vaccination with PBS. Our study revealed that Vac1 resulted in the most favorable growth performance, as well as maintained normal liver and kidney function, mitigating the impact of IBDV infection. Serological analysis using VP2 ELISA kits indicated that Vac1 induced the strongest immunological response among all vaccines. Histopathological examination demonstrated that Vac1 caused minimal lymphoid depletion observed in the lymphoid organs, followed by Vac2. Analysis of cytokine expression profiles showed significant upregulation in all vaccinated groups, particularly Vac1, during the pre-challenge period. Following IBDV infection, Vac1 resulted in a noteworthy increase in the expression of IL2 and IFN-γ, Vac2 showed a significant upregulation in TNF-α and granzyme, and both Vac1 and Vac3 exhibited increased levels of IL1β and IL10. In conclusion, our study suggests that the various vaccines triggered immune responses against IBD through both humoral and cell-mediated immunity. However, recombinant followed by immune complex vaccines appeared to induce more robust immunity while also being safer for broiler chickens in contrast to the intermediate plus vaccine.

## 1. Introduction

Infectious bursal disease, also known as Gumboro disease, is a rapidly spreading acute viral disease affecting young chickens. It is attributed to the infectious bursal disease virus (IBDV) which targets the Fabricius bursa and invades B lymphocytes located within the bursal follicles [1]. IBDV, belonging to the *Birnaviridae* family, imposes significant economic burdens on the poultry sector. It achieves this through the destruction of the bursa, immunosuppression, and high mortality rates in young chickens [2,3,4]. IBDV was firstly detected in the Gumboro province of the United States in 1962 and since then has had a worldwide prevalence and has been characterized by severe immunosuppression in affected birds, and often mortality [5].

Given the economic importance of IBD and its widespread prevalence, vaccination has remained a crucial tool for control [6]. The most used vaccines to control IBD during the last decade included live attenuated vaccines [7]. Commercially available live attenuated vaccines come in various forms, classified according to their level of attenuation as “mild”, “intermediate”, “intermediate plus”, or “virulent” IBD vaccines [8]. Although “intermediate plus” or “hot” vaccines can effectively counteract elevated levels of maternal antibodies, they also carry the risk of causing harm to bursal follicles, leading to immunosuppression [5].

To address the side-effects of live vaccines and the impact of MDA (maternally derived antibodies), different countries have developed and licensed vectored as well as immune complex vaccines [9,10]. The recombinant vaccine employed turkey herpes virus (HVT) as a carrier for the viral protein (VP2) gene of IBDV [11,12]. The immune complex vaccine is produced by blending a live IBDV vaccine strain with IBDV antibodies derived from serum collected from hyperimmunized chickens. This process aims to prevent neutralization by maternal antibodies while decreasing the virulence of the virus [8,13]. These vaccinations might be administered without inducing humoral immunosuppression even when maternal IBD antibodies are present [5], resulting in a delayed virus replication within the bursal tissue and an extended presence of the vaccine virus [14].

Chickens have shown their ability to elicit a cell-mediated cytokine response when confronted with viral infections [15]. In the case of IBDV infection, activated T-cells within the bursa play a vital role in controlling IBDV replication. However, they also contribute to damage and recovery of bursal tissue by releasing proinflammatory cytokines [16]. Key inflammatory cytokines like transforming growth factor-beta (TGF-β), tumor necrosis factor-α (TNF-α), interferon-γ (IFN-γ), and interleukins exhibit elevated expression levels during IBDV infection [17,18,19,20]. Our understanding of the degree of bursal cytokine expression after vaccination, with or without a subsequent challenge, remains limited.

Although there are studies to compare different vaccines [5,10,21,22], this current study provides a complete insight of the assessment of three vaccination strategies against IBD depending on molecular (the gene expression of cytokines associated with the immune response in bursal tissues) and biochemical studies rather than histopathological and serological evaluation. Consequently, we can gain insight into how these vaccines stimulate immune responses and can identify the most suitable vaccination regimen, considering the presence of maternally derived antibodies in broiler chickens on Egyptian farms.

## 2. Materials and Methods

### 2.1. Experimental Design

**Figure 1:**
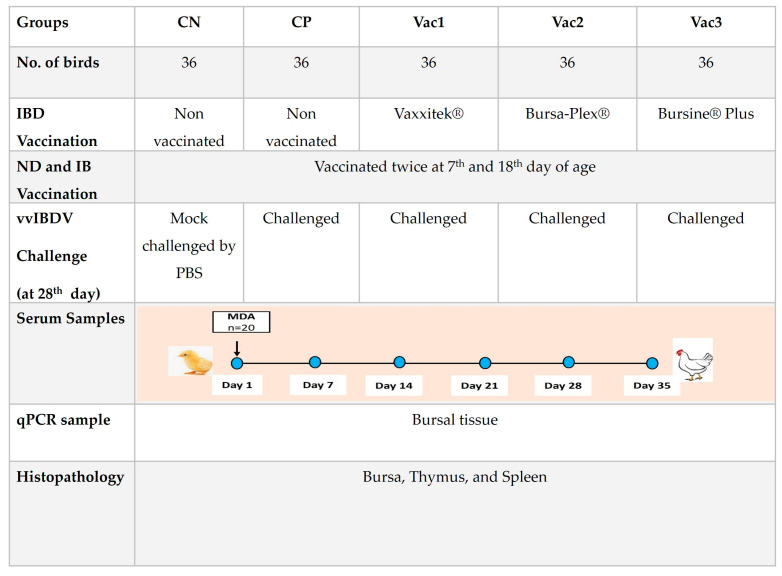
Experimental design shows the experimental groups, vaccination strategies, IBDV challenge, and sampling. CN, control negative; CP, control positive; Vac1, recombinant; Vac2, immune complex; and Vac3, intermediate plus groups.

This experiment was carried out in full compliance with the guidelines and advice provided by the Ethics Committee for Animal Experiments at Kafrelsheikh University, Egypt. (KFS-IACUC/120/2023). We obtained two hundred day-old chicks (Avian 48) from a nearby hatchery (Ayad, Tanta, Gharbeya, Egypt). These chicks were accommodated in clean and well-ventilated floor pens, provided with fresh litter, and housed at the Sakha Research Farm related to the Faculty of Veterinary Medicine at Kafrelsheikh University. Upon arrival, the chicks were kept at a temperature of 33 °C, with a gradual decrease of 1 degree every three days. The chicks had unrestricted access to both feed and water throughout this study. They began with a starter diet, consisting of crumbled feed containing 23% crude protein and with a metabolizable energy (ME) of 3008 Kcal/kg for the initial twenty days. Subsequently, they transitioned to a grower diet, which consisted of pelleted feed with 21% crude protein and ME of 3080 Kcal/kg, during the third week of age. Finally, they were provided with a finisher diet, comprising pelleted feed containing 19% crude protein and with 3190 Kcal/kg ME, until the end of the trial. Out of the total 200 chicks, 20 non-vaccinated day-old chicks were selected for serum collection to detect maternally derived antibodies (MDAs). The remaining 180 chicks were distributed into five sets (*n* = 36). Within each group, there were three replicates, each consisting of twelve birds. These groups consisted of the recombinant (Vac1), immune complex (Vac2), intermediate plus (Vac3), control negative (CN), and control positive (CP) groups. The samples were collected from 3 birds/group every week throughout the five weeks (4 weeks before challenge and 1 week after the challenge) of the experiment. Therefore, 12 birds were used before the challenge and the remaining 24 birds were subjected to IBDV challenge on the 28th day except the CN group. The experimental design is displayed in Figure 1.

### 2.2. IBDV Vaccines and Vaccination Programs

In the present study, three different vaccination programs for IBDV were employed. The first program utilized a recombinant (HVT-IBD vector) vaccine known as Vaxxitek^®^ (Merial S.A.S., Duluth, GA, USA). The development of this vaccine involved the insertion of an IBDV VP2 gene, derived from the Faragher 52/70 IBDV strain, into the genome of HVT. It was administered through subcutaneous injection (S/C) directly at the hatchery (referred to as Vac1). The second vaccination program involved the use of an immune complex (Bursa-Plex^®^) vaccine (Zoetis Inc., New York, NY, USA). This vaccine was also delivered via subcutaneous (S/C) injection to the chicks at the hatchery (referred to as Vac2). The third vaccination program utilized the intermediate plus vaccine “Bursine^®^ Plus” (Zoetis Inc., New York, NY, USA). A single dose of this vaccine was administered through eye drops on the 14th day of age (referred to as Vac3). The control negative (CN) and control positive (CP) groups were not subjected to any IBD vaccinations. Furthermore, all bird groups, including the vaccinated and control groups, were vaccinated against Newcastle Disease (ND) and Infectious Bronchitis (IB) by live attenuated vaccines, Nobilis MA5 + Clone 30^®^ (MSD), at the ages of 7 days and 18 days via the eye drop.

### 2.3. IBD Virus and Challenge

The vvIBDV strain used for the challenge “Banklt2472686 Egypt-IBD-RLQP-2021” was provided by the Reference Laboratory for Veterinary Quality Control on Poultry Production, which is part of the Animal Health Research Institute located in Giza, Egypt. This viral strain was recorded in the gene bank, assigned accession number MZ409478, and was previously used by Ameen et al., 2022 [23]. To assess its infectivity, the virus was titrated in chicken eggs that were specific-pathogen-free (SPF), enabling the determination of the embryo infective dose 50 (EID50) in accordance with the methodology outlined by Aricibasi et al. [24]. Regarding the challenge, a dosage of 10^4^ EID_50_/mL was employed (equivalent to 150 µL per bird, with distribution of 50 µL via the nasal route, 50 µL administered via eye drops, and 50 µL given orally). The challenge was conducted on the remaining birds from each group when they reached 28 days of age, while the CN group was mock-challenged using PBS.

### 2.4. Sampling

The experimental birds were subjected to anesthesia via intraperitoneal injection of 50 mg/kg sodium pentobarbital to ensure their well-being and minimize any potential suffering. Subsequently, the anesthetized broilers were humanely euthanized through cervical dislocation. For the assessment of maternally derived antibody (MDA) titers related to IBDV, twenty one-day-old chicks were utilized, and serum samples were obtained on the first day of their lives. Throughout the duration of this study, blood samples were regularly collected from three birds within each group on a weekly basis, specifically on the 7th, 14th, 21st, 28th, and 35th days of age. These samples were used to obtain serum for ELISA testing. Additionally, small tissue samples were promptly extracted from the bursa, preserved by immersion in liquid nitrogen, and then kept at −80 °C until they were needed for RNA extraction and subsequent qPCR. Histopathological samples were also obtained from the primary lymphoid organs (thymus, spleen, and bursa). These samples were initially preserved in a 10% formalin solution for a 24 h period and then transferred to 70% ethyl alcohol, where they remained until they were ready for histopathological examination.

### 2.5. Clinical Signs, Growth Performance, Mortality, and Postmortem Lesions

Throughout the experimental period, the birds underwent daily observation for any potential clinical signs. To assess the growth performance of the broilers, we calculated various parameters, including IBW (initial body weight), FBW (final body weight), BWG (body weight gain), FI (feed intake), and FCR (feed conversion ratio) within the different experimental groups. Thorough examinations were conducted to identify macroscopic lesions in the dead birds. At the end of the experiment, specifically on the 7th day after IBDV challenge (7dpc), a postmortem (PM) investigation was conducted on all remaining birds as part of the assessment process.

### 2.6. Blood Biochemical and Serological Analysis

Blood samples were obtained from the wing vein of the birds under anesthesia. For the duration of the experiment, we randomly selected three chicks from each group every week, encompassing the 1st, 2nd, 3rd, 4th (pre-challenge), and 5th weeks (7dpc) of age, for serum sample collection. This procedure involved drawing blood into sterile tubes, allowing it to clot at 37 °C for one hour in a sloping position. Subsequently, the tubes were refrigerated overnight, followed by centrifugation at 3000 rpm for a duration of 15 min to isolate the sera. These sera were then preserved at a temperature of −20 °C until further use.

For the biochemical analysis, we utilized commercial kits in conjunction with spectrophotometry to determine ALT (alanine aminotransferase), AST (aspartate aminotransferase), total protein, albumin, creatinine, and uric acid in the serum at the end of the 4th week (pre-challenge) and the 5th week (7dpc).

To assess the IBDV antibody levels, we conducted a serological titration employing two different types of indirect ELISA kits, ID Screen^®^ IBD Indirect (classical kits) and ID Screen^®^ IBD VP2 (VP2 kits for detecting antibodies against IBD VP2 protein), following the manufacturer’s guidelines (ID Vet, Grabels, France). A negative immune status was determined if the ELISA titer was ≤875 (classical kits) or ≤1324 (VP2 kits).

### 2.7. Real-Time PCR (qPCR)

We employed real-time PCR to quantify the immune reaction of the broiler in response to IBD, various vaccines, and the viral challenge. The extraction of total RNA from bursal tissues (2 tissue samples per replicate) was performed. Initially, the tissue was ground using liquid nitrogen, and then RNA isolation was performed using Trizol (iNtRON Biotechnology, Inc., Seongnam, Kyonggi-do, Republic of Korea) in accordance with the manufacturer’s guidelines. The RNA’s integrity was evaluated using 2% agarose gel electrophoresis stained with ethidium bromide. Subsequently, RNA was converted into complementary cDNA using Thermo Scientific cDNA kits (Thermo Fisher Scientific, Branchburg, NJ, USA).

The relative expression of specific cytokines was evaluated. Gene-specific primers (listed in Table 1) were utilized. GAPDH served as the housekeeping gene. Gene amplification was carried out using the Stratagene MX300 P real-time PCR system (Agilent Technologies, Santa Clara, CA, USA) with SensiFast™ SYBR green (Bioline, London, UK). The total reaction volume amounted to 20 μL, consisting of 10 μL of SensiFast™ SYBR master mix, 0.5 μM of primer, and 2 μL of cDNA. The amplification process included an initial pre-denaturation step at 95 °C for 30 s, succeeded by 40 cycles at 95 °C for 10 s, with specific annealing temperatures (specified in Table 1). The fold change was computed using the method established by Livak and Schmittgen [25]. The assessment of RNA integrity, purity, and melting curves for some investigated cytokine genes was shown in Supplementary Figure S1.

### 2.8. Histopathology

Samples from the primary lymphoid organs (bursa, thymus, and spleen) of various experimental groups were collected and preserved in a 10% neutral-buffered formalin solution. After 24 h, the samples were transmitted to 70% ethyl alcohol. The tissue samples were subsequently dehydrated using a series of increasing ethanol concentrations, clarified with xylene, and then impregnated and embedded in paraffin wax. Sections measuring 5 µm were cut using a Leica rotary microtome (Leica Microsystems, Wetzlar, Germany) and placed on glass slides. These prepared tissue sections were deparaffinized with xylene, rehydrated with decreasing concentrations of ethanol, and finally brought to distilled water before undergoing conventional hematoxylin and eosin (H and E) staining, following established procedures [29]. The resulting sections were observed using a light microscope (Leica DM500; Leica Microsystems, Tokyo, Japan).

### 2.9. Statistical Analysis

The collected data, encompassing growth performance, serological, and cytokine gene expression data, underwent checks for normality and homogeneity of distribution using the Shapiro–Wilk and Levene tests, respectively. The results are expressed as means along with their corresponding standard errors. Statistical analysis involved employing analysis of variance (ANOVA) conducted using SPSS 22.0 (SPSS Inc., Chicago, IL, USA). To discern variations among the different treatments, Duncan’s multiple-range test was utilized, with significance set at *p* < 0.05.

## 3. Results

### 3.1. Clinical Signs, Growth Performance, Mortality Rate, and Postmortem Lesions

Three days post challenge, clinical symptoms manifested in the control positive (CP) group, characterized by anorexia, sleepiness, a droopy appearance, watery-whitish diarrhea, and prostration, eventually leading to a mortality rate of 75% (18 out of 24) among the infected birds. In contrast, neither mortality nor abnormal clinical signs were detected in the other vaccinated or CN groups during the trial. The PC broilers that were euthanized displayed marked hemorrhages observed in the muscles of the chest and thigh, as well as an enlarged bursa, thymus, spleen, and kidneys.

The mean values for growth performance parameters including IBW, FBW, BWG, FI, and FCR are presented in Table 2. Both Vac1 and Vac2 groups exhibited a noteworthy rise (*p* ≤ 0.05) in FBW and BWG, followed by CN and Vac3 groups, while CP demonstrated the lowest values. Concerning FI, the CP group exhibited a significantly higher value, whereas CN had a significantly lower value. FCR was meaningly higher in the CP group, followed by Vac3, while CN, Vac1, and Vac2 displayed significantly lower values (*p* ≤ 0.05).

### 3.2. Biochemical Analysis

The statistical analysis of biochemical parameters (Table 3) indicated significant elevated AST and ALT levels in the CP group after IBDV challenge. Conversely, the CN group exhibited a reduction in AST and ALT levels. However, the Vac3 group showed statistically increased AST levels than the CN group before and after the challenge, which is a numerical but not significant increase compared to other vaccinated groups. Also, the Vac3 group displayed statistically increased ALT levels than only the Vac2 group before and after the challenge. Regarding the uric acid levels, there was a significant decrease in the control as well as Vac3 groups before the challenge, while Vac1 and Vac2 showed a significant increase. After IBDV challenge, the CP as well as other vaccinated groups exhibited a significant increase in the uric acid level. As for other parameters, including total protein, albumin, globulin, and creatinine, there were no significant differences observed among all groups, neither before nor after the IBDV challenge.

### 3.3. IBDV Antibody Titer

**Figure 2:**
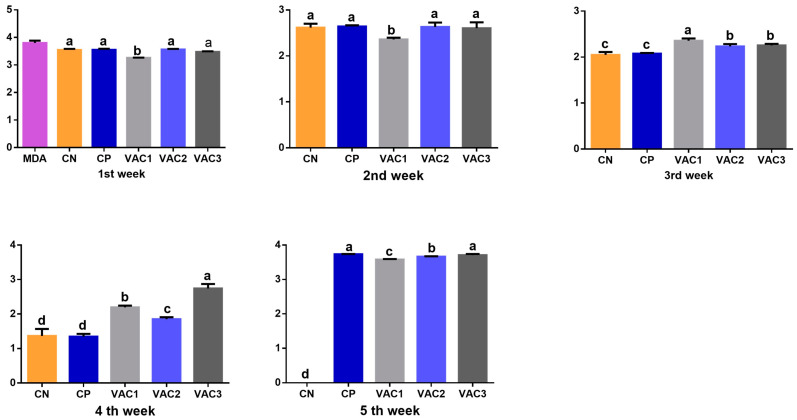
Analysis of IBDV antibody titers using Classical ELISA kits in broiler chickens vaccinated with recombinant vaccine (Vac1), immune complex (Vac2) at hatchery, and intermediate plus (Vac3) at 14 days. MDA, maternally derived antibodies on 1st day of age. CP, control positive group. All groups subjected to IBDV challenge on the 28th day of age except CN (BPS). Different superscript letters at the same time indicate significant differences between groups (*p* < 0.05) using one-way ANOVA.

Maternally derived antibodies (MDAs) against IBDV were initially detected in one-day-old chicks, with an average titer of 6050 ± 100 using classical kits and 12,969 ± 100 using VP2 kits. According to the ELISA test instructions, an ELISA titer below 875 (for classical kits) would indicate a negative IBDV immune status. Therefore, all groups exhibited negative antibody titers starting from the second week of age. Notably, the Vac1 group had the lowest titer at 229 ± 13 (Figure 2).

**Figure 3:**
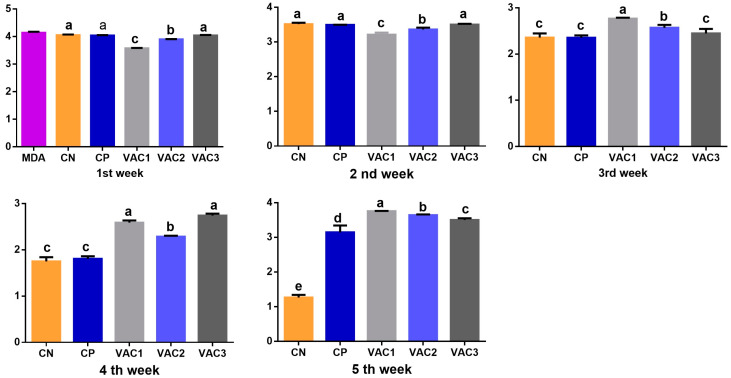
Analysis of IBDV antibody titers using VP2 ELISA kits in broiler chickens vaccinated with recombinant vaccine (Vac1), immune complex (Vac2) at hatchery, and intermediate plus (Vac3) at 14 days. MDA, maternally derived antibodies on 1st day of age. CP, control positive group. All groups subjected to IBDV challenge on the 28th day of age except CN (BPS). Different superscript letters at the same time indicate significant differences between groups (*p* < 0.05) using one-way ANOVA.

Regarding the VP2 kits (Figure 3), an ELISA titer less than 1324 was considered negative. Consequently, the IBDV antibody titers were negative from the 3rd week until the time of IBDV challenge in all experimental groups. However, both the non-vaccinated control (230 ± 28) and the Vac3 group (286 ± 38) displayed the lowest values.

One week post challenge, all challenged groups exhibited significantly positive IBDV antibody titers. Among them, the CP group had the highest value compared to the other groups. For the other vaccinated broilers, there was a notable increase in titers, with Vac3 showing the highest increase followed by Vac2 and then Vac1 when using classical kits (Figure 2). Conversely, with VP2 kits (Figure 3), the CP group displayed the lowest value, while the other vaccinated groups demonstrated a significant increase, with Vac1, followed by Vac2 and then Vac3.

### 3.4. Cytokine Gene Expression

**Figure 4:**
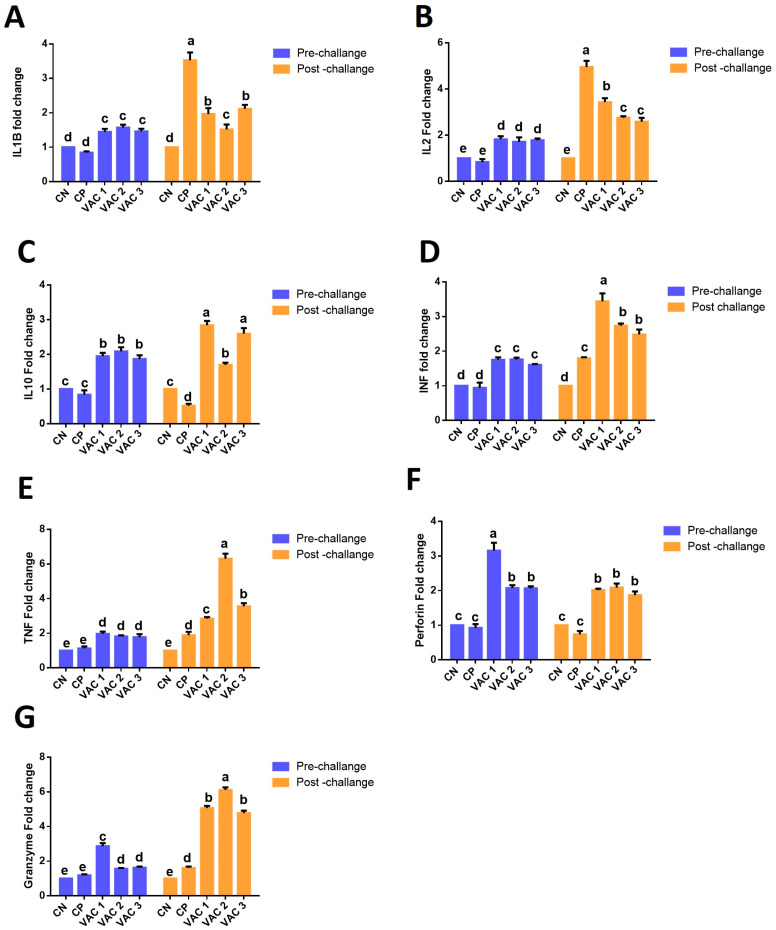
The modulatory effect of recombinant (Vac1), immune complex (Vac2) at hatchery, and intermediate plus vaccine (Vac3) on the relative expression of cytokine genes in broiler chickens’ bursal tissues pre- and post-vvIBD challenge. (**A**) IL1β, (**B**) IL2, (**C**) IL10, (**D**) IFN, (**E**) TNF, (**F**) perforin, and (**G**) granzyme. The relative expression as a fold change during pre- (28th day) and post-challenge (7dpc). The different letters indicate significant differences (*p* < 0.05). Data were normalized against GAPDH housekeeping gene and are expressed as means ± SE.

The three vaccines employed in this study significantly influenced the expression of cytokine genes during the pre-infection period compared to the control group. Furthermore, the viral challenge had a more pronounced effect on cytokine expression in the control positive (CP) group and vaccinated groups than in the pre-challenge samples, as depicted in Figure 4.

During the pre-challenge period, all vaccines led to a significant upregulation (*p* < 0.05) of cytokines including IL1β, IL2, IL10, IFN-γ, and TNF-α (Figure 4A–E) in comparison to the control groups. Notably, the expression levels of these cytokines were relatively similar across the different vaccinated groups (*p* > 0.05). As for perforin and granzyme relative expression (Figure 4F–G), all vaccines significantly increased their expression in comparison to the control group, with Vaxxitek (Vac1) exhibiting the highest expression (*p* > 0.05) relative to Bursa-Plex (Vac2) and Bursine plus (Vac3) vaccines.

Following the IBDV challenge, cytokine expression varied. The control positive group (CP) displayed the highest expression of pro-inflammatory cytokines, specifically IL1β and IL2 (Figure 4A,B). In contrast, CP exhibited a significant downregulation of IL10 (Figure 4C) in comparison to the other groups. Regarding perforin (Figure 4F), there was no significant difference observed between CP and CN. The IBDV challenge induced a significant upregulation (*p* < 0.05) of IFN-γ, TNF-α, and granzyme (Figure 4D,E,G, respectively) in the CP group. Nonetheless, these levels remained lower than those observed in other vaccinated broilers.

The post-challenge expression of cytokines in the vaccinated groups exhibited different levels and kinetics. All vaccinated groups displayed an upregulation of cytokine expression after the challenge, with variations in the levels of expression. Specifically, IL1β and IL10 (Figure 4A,C) exhibited a significant increase in Vac1 and Vac3 compared to Vac2. The pro-inflammatory genes Il2 and IFN-γ (Figure 4B,D) exhibited a significant increase in Vac1 when compared to Vac2 and Vac3. TNF and granzyme (Figure 4E,G) displayed a noticeable rise in Vac2 compared to Vac1 and Vac3. In contrast, perforin (Figure 4F) showed an upregulation without any significant difference among the vaccinated birds.

### 3.5. Histopathology

#### 3.5.1. Bursa

**Figure 5:**
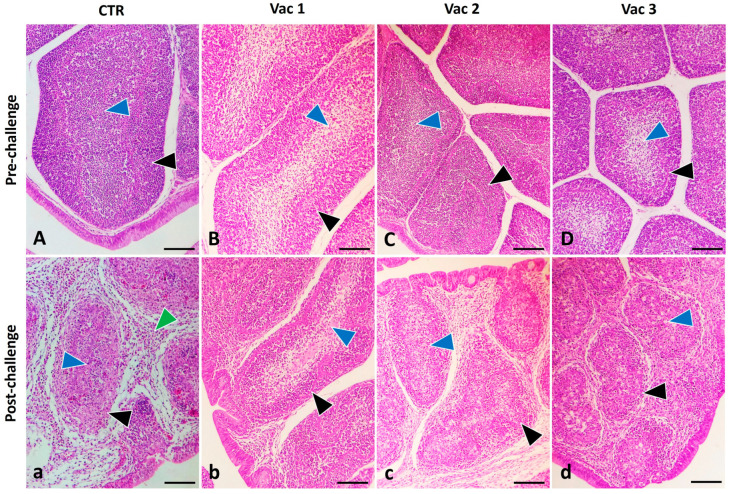
Photomicrograph of broiler chickens’ bursa in the control group (CTR) as well as other vaccinated groups including Vaxxitek (Vac1), Bursa-Plex (Vac2), and live attenuated (Vac3) during pre-challenge (**A**–**D**) and post-challenge (**a**–**d**) period. The post-challenged CTR group showed severe necrotic changes (blue arrowhead) of the bursal compartments with massive infiltration of inflammatory cells (green arrowhead), while the bursa of Vac1- and 2-vaccinated groups revealed normal appearance like that of the control ones that exposed normal cortex (black arrowhead) and medulla (blue arrowhead). Post-challenged Vac3 revealed less condensation of the lymphocytes in the cortex (black arrowhead) and necrosis with histiocytic infiltration in the medulla (blue arrowhead). Stain H&E. Bar 100 µm.

In pre-challenged birds, the bursal follicles exhibited a characteristic arrangement characterized by a densely stained outer cortex primarily composed of tightly packed lymphocytes and a lighter stained inner medulla containing fewer cells of varying sizes. These bursal follicles were divided by connective tissue fibers that contained blood vessels and a limited number of cells (Figure 5A–C). The general structure of the bursal follicles in the Vac3 group closely resembled that of the CN group, albeit with lower cellularity in the medulla or lymphoid depletion (Figure 5D). Post challenge, birds exhibited severe necrotic changes in the bursal compartments, marked by a massive infiltration of inflammatory cells and poorly differentiated bursal follicles into cortex and medulla (Figure 5a). In contrast, the bursa of Vac1- and Vac2-treated groups returned to a normal appearance, like the control group (Figure 5b,c), although in the Vac3 group, there was less condensation of lymphocytes in the cortex and some necrosis with histiocytic infiltration in the medulla (Figure 5d).

#### 3.5.2. Thymus

**Figure 6:**
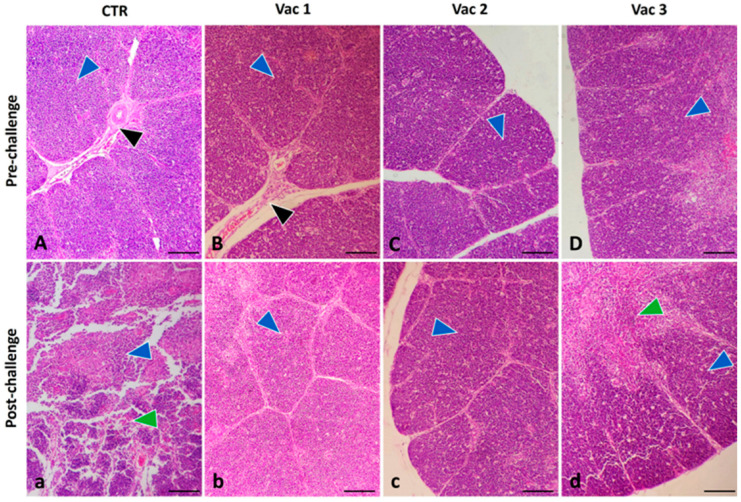
Photomicrograph of broiler chickens’ thymus in the control group (CTR) as well as other vaccinated groups including Vaxxitek (Vac1), Bursa-Plex (Vac2), and live attenuated (Vac3) groups during pre-challenge (**A**–**D**) (upper panel) and post-challenge (**a**–**d**) (lower panel) period. The thymic compartments (blue arrowhead) were almost normal, intact, and separated by interstitial connective tissue (black post arrowhead) before challenge in all groups in addition to Vac1 and Vac2 post-challenged groups. The challenged CTR group (**a**) demonstrated severe necrosis (green arrowhead) with massive mononuclear inflammatory cell infiltration in the necrosed compartments (blue arrowhead). Vac3 (**d**) showed small thymic compartments (blue arrowhead) with infiltration of inflammatory cells and mucosal microcyst formation (green arrowhead) within the interstitial areas. Stain H&E. Bar 100 µm.

In pre-challenged birds, the thymic compartments featured an outer cortex with deeply stained, numerous small lymphocytes possessing large nuclei. In contrast, the central medulla exhibited a lighter stain and contained numerous epithelial cells with large, pale nuclei, along with a smaller population of lymphocytes (Figure 6A). These divisions within the thymus were isolated by interstitial connective tissue partitions (Figure 6A–C), although there was less cellularity in the medulla of both groups (Figure 6D). After the challenge, the thymic structure in the CP group displayed extensive necrosis, accompanied by a substantial influx of inflammatory cells into the necrotic areas. This resulted in complete disruption of the typical morphology of the thymus (Figure 6a). The thymic compartments in the Vac1 and Vac2 groups were almost normal like the pre-challenged groups (Figure 6b,c). On the contrary, the thymic compartments in the Vac3 (Figure 6d) group were small, with interstitial areas occupied by inflammatory cells and mucosal microcyst formation within the interstitial areas.

#### 3.5.3. Spleen

**Figure 7:**
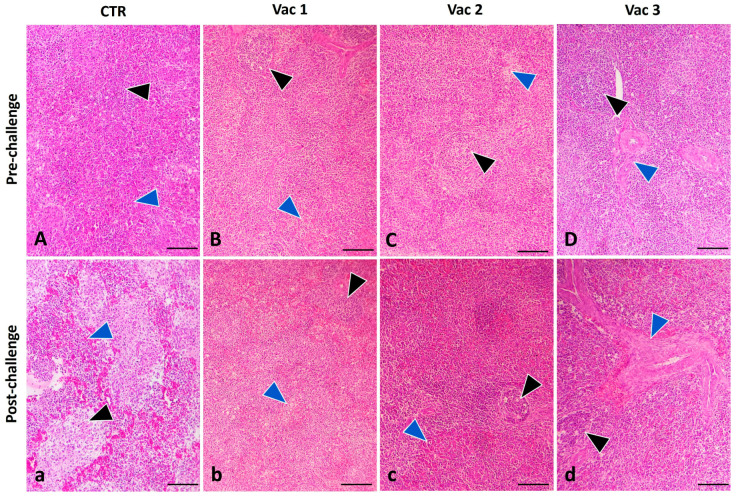
Photomicrograph of broiler chickens’ spleen in the control group (CTR) as well as other vaccinated groups including Vaxxitek (Vac1), Bursa-Plex (Vac2), and live attenuated (Vac3) groups during pre-challenge (**A**–**D**) (upper panel) and post-challenge period (**a**–**d**) (lower panel). The post-challenged CTR birds displayed severely affected spleen with marked lymphoid depletion and necrosis (black arrowhead), massive histiocytic infiltration (blue arrowhead). Vac1, 2 (**b**,**c**) showed nearly similar morphology like the control group, respectively. Vac3 (**d**) revealed necrosis and histiocytic infiltration in the red pulp (blue arrowhead) and decreased condensation of the lymphocytes in the white pulp (black arrowhead). Stain H&E. Bar 100 µm.

In pre-challenged birds, the spleen displayed well-defined red and white pulp regions. The white pulp contained lymphocytes of various sizes as well as plasma cells, all accompanied by intact arterioles. The red pulp, on the other hand, consisted of venous sinuses and various types of cells (Figure 7A), with slight follicular cell depletion (Figure 7B–D). Post challenge, severely affected spleens exhibited a significant depletion of lymphoid tissue, necrosis, extensive histiocytic infiltration, and a complete absence of the typical architectural structure of the splenic compartments (Figure 7a). In contrast, post challenge, spleens in the Vac1 and Vac2 groups displayed a morphology that closely resembled that of the CN group (Figure 7b,c), while the Vac 3 group showed necrosis and histiocytic infiltration in the red pulp and a reduced condensation of lymphocytes in the white pulp (Figure 7d).

## 4. Discussion

The present work focused on assessing the effectiveness of three different IBD vaccination schemes in commercial broilers with elevated MDA. This assessment was based on various criteria, including clinical observations, the feed conversion ratio (FCR), mortality rates, serological analysis, cytokine genes expression, as well as histopathological findings.

Typically, clinical disease resulting from IBDV infection can be identified through a combination of distinct clinical symptoms and postmortem observations [30]. Throughout the pre-challenge phase, none of the experimental groups exhibited mortality or unusual clinical symptoms. Following the vvIBD challenge, only broilers in the PC group displayed symptoms such as reduced appetite, lethargy, a drooping posture, watery-whitish diarrhea, and weakness, ultimately resulting in a 75% mortality rate. Postmortem examinations revealed enlarged lymphoid organs including the bursa (BF), thymus, spleen, as well as kidneys, along with hemorrhages in the thigh and pectoral muscles. On the contrary, the remaining vaccinated birds remained healthy, with no mortality or postmortem findings indicative of lesions. These finding agree with previous investigations [5,10].

Throughout the study, we assessed the growth performance of all groups. Vac1 demonstrated the most favorable results in terms of FBW and BWG. Conversely, the CN group exhibited the lowest feed conversion ratio (FCR), followed by Vac2 and Vac1. These results are similar to previous findings [10,31].

The biochemical study exposed that Vac3 slightly increased the hepatic function where it exhibited increased levels of AST and ALT than those of the CN group, both prior to and following the challenge, resembling the observations in the CP group. However, there was no significant difference between different vaccines. The elevated AST and ALT could be attributed to either viral replication in the birds vaccinated with the intermediate plus vaccine, as suggested by [32], or it may be a result of the stress induced by vaccination, as discussed by [22]. Additionally, uric acid levels showed a significant increase in Vac1- and Vac2-vaccinated birds, before the challenge as well as all vaccinated groups after the IBDV challenge. These findings indicate that there was no significant difference between different vaccines on the liver and kidney function of immunized broilers. Furthermore, no significant variations were observed in other biochemical parameters, as previously reported by [22].

When assessing antibody levels against Gumboro disease, it is important to note that there are two distinct ELISA kits employed for this purpose: the classical and VP2 kits. The key difference between these kits lies in the specific antigens they target for detection. The findings from this current study showed a notable increase in MDA that was determined by both types of ELISA kits when the birds were one week old. The broiler chicks were hatched with elevated maternal antibodies, as previously observed [10,33]. All immunized birds showed a significant decrease in ELISA antibody titer up to the day of challenge that may be due to the interference of MDA or an insufficient immune reaction [34,35,36]. These MDA levels were considered negative for all groups from the second week of age, particularly the Vac1 group. The rapid and significant decrease in the MDA in the Vac1 group compared to the other groups may be due to the immunosuppressive or stress effect of HVT during the early stage after immunization. Typically, MDA levels in chicks decline by 21 days of age, which is consistent with the usual timeframe for the MDA decline within 2 to 3 weeks post hatching, as reported by [37,38].

The levels of IBDV antibodies were assessed through conventional ELISA kits, affirming previous results as outlined in the described method by [39,40], which indicated that DNA vaccines result in relatively low ELISA antibody titers. Remarkably, the limited seroconversion observed in the recombinant vaccine group after the vvIBDV challenge resembled the outcomes previously documented [41]. This might be attributed to the presence of high antibody titers that neutralized a portion of the challenged virus. Consequently, the traditional ELISA kits employed for detecting antibodies may not accurately measure the antibodies produced by the recombinant HVT-VP2 vaccine [10]. On the other hand, VP2 ELISA kits are more specific and revealed a significant increase in antibody titer for Vac1 against IBDV 7dpc more than Vac2 and Vac2 vaccines.

The suboptimal immune response to the immune complex vaccine, observed from the second or third week onward as per the ELISA Kit manufacturer’s guidelines until the time of IBDV infection, could be attributed to the presence of virus-neutralizing factors that continue to bind to the vaccine virus [42,43]. The intermediate plus vaccine initially displayed negative results, but it exhibited a significant increase on the 28th day (2 weeks after its vaccination); however, it was still within the negative value. These findings agreed with the investigation of Wyeth and Chettle [44], who reported that the immune response to intermediate plus vaccines in broilers with substantial maternal antibodies might typically be anticipated around eighteen days after immunization. This may confirm that the intermediate vaccines were unable to overcome the existence of high maternal antibodies [34,45].

Upon exposing the chickens to vvIBDV when they were four weeks old, they displayed seroconversion, evidenced by elevated ELISA antibody titers in the challenged birds in comparison to the non-challenged groups. This suggests that the ELISA antibody titer alone might not serve as the sole indicator of the birds’ capacity to resist the vvIBDV infection [10]. Furthermore, it is important to recognize that ELISA kits provide quantitative rather than qualitative data on the birds’ immune status. Therefore, it is crucial to ascertain the specificity of antibodies. In this regard, VP2 ELISA kits offer greater specificity. Previous studies indicated that the effectiveness of the recombinant HVT vector vaccine against severe IBDV challenge is positively correlated with the degree of VP2 antigen expression within the vaccine [12,46]. However, the vaccine’s efficacy was only partial and exhibited a restricted duration [47,48]. In the current study, Vac1 exhibited the most elevated antibody titers 7 days after the challenge when using VP2 ELISA kits. This may be interpreted as the initial vaccination serving as a prime (eliciting low antibody titers but establishing memory B cells), while the challenge served to boost high titer antibody production that was significantly increased than other vaccinated groups. Nevertheless, additional investigations may be required to assess the vaccine’s efficacy over an extended period following the challenge.

The ability of a virus to elicit non-B cell immunity could hold significance in providing protection and establishing enduring immunological memory, as noted by [49]. Sustained antigenic stimulation may potentially contribute to the more effective maintenance of T cell-mediated immune responses, as suggested by [50]. A robust immune reaction against IBDV is crucial for safeguarding against the aggressive virus. Nevertheless, it is widely acknowledged that relying solely on antibodies is insufficient to ensure comprehensive protection [51]. During the early phases of viral infection, T lymphocytes promptly invade the bursa of Fabricius [52], suggesting their probable involvement in the host’s defense mechanism. Cytokines, acting as essential mediators and regulators of host responses to foreign antigens, have a primary function in coordinating and controlling the operational activities of immune cells [27]. During the pre-challenge period, all investigated cytokines were significantly upregulated in all vaccinated groups. Interestingly, the gene expression related to cytolytic cytokines, specifically perforin and granzyme, was significantly increased in Vac1-vaccinated birds compared to other groups. These results suggested that the cytotoxic cytokines are more indicative for a rapid and protective immune response induced by Vac1 to enhance T-cell responses in conjunction with antibody responses.

IBDV infection triggered a notable increase in cytokine expression where the results indicated a substantial upregulation in the expression of both proinflammatory and cytotoxic cytokines upon challenge with vvIBDV at 7 days post challenge (7dpc). After the viral challenge, the cytokine expression was variable. The pro-inflammatory cytokines IL1β and IL2 showed the highest expression (*p* < 0.05) in the control positive group in relation to all the other groups. This observation implies that activated T cells have the potential to participate in antiviral defense and may play a part in the removal of the IBD virus [51]. On the other hand, the CP birds revealed a significant downregulation for IL10 (*p* < 0.05). Related to perforin, there was no noteworthy distinction observed between the CP and CN groups. The viral challenge induced a significant upregulation of IFN-γ, TNF-α, and granzyme in the CP group; however, it was still lower than other vaccinated groups. These results indicate the role of IBD vaccines in the modulation of these cytokines within the bursal tissue.

The post-challenge expression of cytokines in the vaccinated groups showed different levels and kinetics. All vaccinated groups showed a post-challenge upregulation with different levels of cytokine expression. It is worth noting that changes in cytokine levels are a fundamental aspect of the immune reaction of birds when combating infections [53]. This is consistent with a prior study that explained how IBDV infection led to varying elevations in different cytokines in conjunction with changes in IBDV proliferation in the bursal tissue [20,27]. The current study elucidated that Vac1 and Vac3 caused a significant rise in IL1 β and IL10 compared to Vac2. On the other hand, El-Fetouh et al. [21] found that the expression level of IL-1β was notably higher in IBDV-infected birds following Vac2 vaccination compared to the challenged birds following intermediate plus vaccination. The upregulation of IL-1β aligns with the cytokine profile previously observed in cases of virulent IBDV infection [54].

The pro-inflammatory genes Il-2 and IFN-γ revealed a significant increase in Vac1 than Vac2 and Vac3. IL-2, in conjunction with various other cytokines such as IL-7, IL-9, IL-15, and IL-21, assumes a vital function in the growth of B cells and the distinction of T cells into effector and memory cells [55]. The upregulation of Il2 and IFN-γ in the Vac1-immunized birds could be attributed to the active recovery of the bursa after the challenge. Furthermore, elevated IL-2 levels might confer an advantage in generating a more robust immune reaction, as evidenced by reasonably elevated antibody titers in comparison with the Vac2 vaccine [21].

TNF-α and granzyme revealed a significant increase in Vac2 than Vac1 and Vac3. While perforin showed upregulation in all vaccinated groups. TNF-α has been recognized as a key regulator of inflammatory reactions and plays an essential role in a typical immune response [56], and its increased level is correlated with alterations in the proliferation of bursal tissue [20,27]. Moreover, cytotoxic T cells perform the eradication of virus-infected cells by releasing lytic proteins, mainly perforin and granzymes. These proteins are discharged through exocytosis when virus-infected targets are identified [57,58]. Notably, the level of perforin in the present study showed a noteworthy increase in all immunized birds, with similar levels of expression. The elevated levels of cytolytic proteins, specifically perforin and granzyme within bursal tissue, along with perforin production by CD4^+^ and CD8^+^ T cells strongly suggest the contribution of these molecules in cytotoxic mechanisms that participate in the clearance of IBDV [52]. On the other hand, the exceptional reduction in perforin expression from the pre- to post-challenge Vac1 group could be due to immune system modulation. The virus may manipulate the host’s immune response, leading to a reduced expression of perforin. It might disrupt the normal functioning of immune cells responsible for perforin production.

To confirm the diagnosis of IBD, it is essential to complement it with histopathological analysis [59]. The pathogenic effects of IBDV extended to lymphoid organs beyond the bursa, including the spleen and thymus [38]. However, Vac1 followed by Vac2 groups displayed the least degree of bursal atrophy. This observation may be due to the correlation between the initiation of humoral immunity, bursal damage, and IBDV multiplication [50]. Likewise, the Vac1 group displayed the least severe histopathological lesion score in the thymus and spleen following the challenge. All these outcomes highlight the superiority of Vac1 in providing defense against the vvIBDV infection, consistent with the previous studies [31,33,60]. In the same context, there was an obvious lymphoid depletion in the lymphoid organs in the live attenuated group [61] that became more severe after viral challenge.

## 5. Conclusions

Our study indicates that the investigated vaccines stimulated immune responses via both humoral and cell-mediated pathways. VP2 ELISA tests showed that Vac1 had the strongest immune response post challenge. Cytokine expression profiling revealed upregulation in all vaccine groups, particularly Vac1, before the challenge. After IBDV infection, Vac1 increased IL2 and IFN-γ expression, Vac2 upregulated TNF-α and granzyme, and both Vac1 and Vac3 had higher IL1β and IL10 levels. Vac1 also caused minimal lymphoid depletion, followed by Vac2, according to histopathological examination. Collectively, the recombinant as well as immune complex vaccines appeared to offer robust immunity while being safer for broiler chickens compared to the intermediate plus vaccine.

### Limitations of this Study

We conducted the present study on the immunological response through three vaccination strategies against IBDV disease from different points of view including serological, cytokines’ expression profiling, and histopathological examination. However, there were some potential limitations that should be considered due to restricted resources and the absence of funding projects. These constraints include short timeframes which limited the ability to capture long-term immunological responses particularly after challenge. Also, different administration times and routes of vaccines were used, where Vac1 and Vac2 were administered on the 1st day via S/c injection while Vac3 was administered on the 14th day via eye drop to simulate the same strategies already used in the farms. Furthermore, limited advanced immunological assays and techniques that require expensive equipment and reagents such as the virus-neutralization test, ELISPOT, lymphocyte proliferation assay, or cytokine protein level detection align with cytokine expression profiling. We recommend these further investigations to support the findings of the current study and to give more comprehensive knowledge about the immunological response against IBDV disease.

## Figures and Tables

**Table 1:** Primers of investigated genes with annealing temperature, accession number, and reference for each gene.

Gene	Primer Sequence	Annealing Tm	Accession NO/Reference
IFN-γ	F: TGCCTGCAGAAGAAGCCTCG R: GACGGGCTCAAAAACCTCCT	60	FJ977575.1 [26]
TNF-α	F: TTCTGGGACCACTGTATGCTCTT R:TACCGACAAAGTGAGAATCAATCAG	60	MF000729.1 [26]
Granzyme A	F: CAGACCAGCACCAGTCATCA R: TCCCGTTCTCATCCATCTTCTC	60	NM_204457.1 [26]
Perforin	F: CTCCCGATGAACGACTTGAG R: CTGAGACTGGCTCCTTTTCC	60	KC551799.1 [26]
Il 2	F: CGCTCAGAACGACGTCAA R: GTCGTCCACACCAACGAG	60	AF000631.1 [27]
IL10	F: CACCCGCACCAAAAGATGAAG R: CGCCTCCTGGAAAACACACAAC	62	NM_001004414.2 [28]
IL1B	F: CCTGATACTTCCTGGAGATTTGTGC R: TTTTTGTCCGTGAATGGGCTC	62	NM_204524.1 [28]
GAPDH	F: TGCCATCACAGCCACACAGAAG R: ACTTTCCCCACAGCCTTAGCAG	60	AF047874.1 [27]

**Table 2:** Effect of different IBDV vaccines on the performance parameters of broiler chickens. IBW, initial body weight. FBW, final body weight. BWG, body weight gain. FI, feed intake. FCR, feed conversion ratio. CN, control negative group. CP, control positive group. Vac1, Vaxxitek. Vac2, Bursa Plex. Vac3, intermediate plus vaccine. Values are means ± standard error. The different small letters indicate significant differences (*p* < 0.05) in the same row.

	CN	CP	Vac1	Vac2	Vac3
IBW	41.00 ± 1.00	41.33 ± 0.88	41.00 ± 1.00	40.33 ± 0.88	40.67 ± 0.33
FBW	1910 ± 55.68 ^b^	1610 ± 32.15 ^c^	2170 ± 36.06 ^a^	2280 ± 52.92 ^a^	1900 ± 20.82 ^b^
BWG	1869.00 ± 55.33 ^b^	1568.67 ± 31.48 ^c^	2129.00 ± 37.03 ^a^	2239.67 ± 52.79 ^a^	1859.33 ± 21.14 ^b^
FI	2878.00 ± 91.77 ^c^	3839.00 ± 114.53 ^a^	3525.33 ± 89.29 ^ab^	3686.33 ± 93.45 ^ab^	3473.67 ± 107.11 ^b^
FCR	1.54 ± 0.012 ^c^	2.45 ± 0.029 ^a^	1.66 ± 0.044 ^c^	1.65 ± 0.032 ^c^	1.87 ± 0.078 ^b^

**Table 3:** Biochemical analysis in different experimental groups including CN (control negative), CP (control positive), Vac1 (Vaxxitek), Vac2 (Bursa Plex), and Vac3 (intermediate plus) vaccines during pre-challenge and post-challenge. The different small letters indicate significant differences (*p* < 0.05) in the same row, while the different capital letters indicate significant differences (*p* < 0.05) between pre-and post-challenge within the same parameter.

Parameter	Time	CN	CP	Vac1	Vac2	Vac3
AST	Pre-challenge	15.6 ± 1.15 ^b^	16.6 ± 1.15 ^Bab^	19.66 ± 3.05 ^ab^	19.33 ± 0.57 ^ab^	21.66 ± 9.07 ^a^
	Post-challenge	16 ± 2 ^b^	25 ± 2.5 ^Aa^	20 ± 1.5 ^ab^	22.6 ± 3.5 ^ab^	26 ± 4.35 ^a^
ALT	Pre-challenge	21.66 ± 5.68 ^abc^	22 ± 1 ^Bab^	20.33 ± 2.5 ^abc^	17 ± 6.24 ^c^	23.33 ± 5.85 ^a^
	Post-challenge	21 ± 3.46 ^abc^	28.66 ± 3.41 ^Aa^	21.33 ± 3.21 ^abc^	19 ± 1 ^c^	27.66 ± 2.5 ^a^
Total Protein	Pre-challenge	3.85 ± 0.92	3.9 ± 0.01	3.3 ± 0.85	3.6 ± 0.01	4.2 ± 0.56
	Post-challenge	3.5 ± 0.01	4.4 ± 3.12	3.7 ± 0.01	3.1 ± 0.56	4.1 ± 0.01
Albumin	Pre-challenge	1.09 ± 0.13	1.35 ± 0.2	1.17 ± 0.07	1.36 ± 0.18	1.22 ± 0.27
	Post-challenge	1.14 ± 0.12	1.25 ± 0.25	1.2 ± 0.08	1.19 ± 0.28	1.5 ± 0.19
Globulin	Pre-challenge	2.47 ± 0.75	3.01 ± 0.45	2.09 ± 0.61	2.47 ± 0.48	2.5 ± 0.62
	Post-challenge	2.42 ± 0.51	2.97 ± 1.28	2.29 ± 0.18	2.14 ± 0.3	2.6 ± 0.44
Creatinine	Pre-challenge	0.51 ± 0.1	0.55 ± 0.08	0.48 ± 0.11	0.5 ± 0.11	0.55 ± 0.03
	Post-challenge	0.53 ± 0.1	0.52 ± 0.03	0.52 ± 0.14	0.6 ± 0.1	0.64 ± 0.08
Uric Acid	Pre-challenge	7.96 ± 1.68 ^b^	7.46 ± 1.76 ^Bb^	10.16 ± 2.26 ^a^	9.5 ± 0.6 ^a^	8.43 ± 0.7 ^Bb^
	Post-challenge	8.2 ± 1.15 ^b^	11.63 ± 2.9 ^Aa^	10.43 ± 1.96 ^a^	10.4 ± 0.95 ^a^	11.26 ± 1.1 ^Aa^

## Data Availability

The data are available from the corresponding author on reasonable request.

## Supplementary Materials

The following supporting information can be downloaded at https://www.mdpi.com/article/10.3390/vaccines12010027/s1, Figure S1: Assessment of RNA integrity, purity, and melting curves for some investigated cytokine genes.

## References

[1] Roh J.-H., Kang M., Wei B., Yoon R.-H., Seo H.-S., Bahng J.-Y., Kwon J.-T., Cha S.-Y., Jang H.-K. (2016). Efficacy of HVT-IBD vector vaccine compared to attenuated live vaccine using in-ovo vaccination against a Korean very virulent IBDV in commercial broiler chickens. Poult. Sci..

[2] Kibenge F.S., Dhillon A., Russell R. (1988). Biochemistry and immunology of infectious bursal disease virus. J. Gen. Virol..

[3] Lukert P., Saif Y., Calnek B.W. (1997). Infectious bursal disease virus. Disease of Poultry.

[4] Berg T.P.V.D. (2000). Acute infectious bursal disease in poultry: A review. Avian Pathol..

[5] Prandini F., Simon B., Jung A., Pöppel M., Lemiere S., Rautenschlein S. (2016). Comparison of infectious bursal disease live vaccines and a HVT-IBD vector vaccine and their effects on the immune system of commercial layer pullets. Avian Pathol..

[6] Van den Berg T., Morales D., Eterradossi N., Rivallan G., Toquin D., Raue R., Zierenberg K., Zhang M., Zhu Y., Wang C. (2004). Assessment of genetic, antigenic and pathotypic criteria for the characterization of IBDV strains. Avian Pathol..

[7] Kannaki T., Priyanka E., Abhilash M., Haunshi S. (2021). Co-administration of toll-like receptor (TLR)-3 agonist Poly I: C with different infectious bursal disease (IBD) vaccines improves IBD specific immune response in chicken. Vet. Res. Commun..

[8] Müller H., Mundt E., Eterradossi N., Islam M.R. (2012). Current status of vaccines against infectious bursal disease. Avian Pathol..

[9] Taghavian O., Spiegel H., Hauck R., Hafez H.M., Fischer R., Schillberg S. (2013). Protective oral vaccination against infectious bursal disease virus using the major viral antigenic protein VP2 produced in Pichia pastoris. PLoS ONE.

[10] Sedeik M.E., El-Shall N.A., Awad A.M., Abd El-Hack M.E., Alowaimer A.N., Swelum A.A. (2019). Comparative evaluation of HVT-IBD vector, immune complex, and live IBD vaccines against vvIBDV in commercial broiler chickens with high maternally derived antibodies. Animals.

[11] Bublot M., Pritchard N., Le Gros F.-X., Goutebroze S. (2007). Use of a vectored vaccine against infectious bursal disease of chickens in the face of high-titred maternally derived antibody. J. Comp. Pathol..

[12] Darteil R., Bublot M., Laplace E., Bouquet J.-F., Audonnet J.-C., Rivière M. (1995). Herpesvirus of turkey recombinant viruses expressing infectious bursal disease virus (IBDV) VP2 immunogen induce protection against an IBDV virulent challenge in chickens. Virology.

[13] Ignjatovic J., Gould G., Trinidad L., Sapats S. (2006). Chicken recombinant antibodies against infectious bursal disease virus are able to form antibody–virus immune complex. Avian Pathol..

[14] Dey S., Pathak D.C., Ramamurthy N., Maity H.K., Chellappa M.M. (2019). Infectious bursal disease virus in chickens: Prevalence, impact, and management strategies. Vet. Med. Res. Rep..

[15] Degen W.G., van Daal N., Rothwell L., Kaiser P., Schijns V.E. (2005). Th1/Th2 polarization by viral and helminth infection in birds. Vet. Microbiol..

[16] Rautenschlein S., Yeh H.-Y., Sharma J. (2002). The role of T cells in protection by an inactivated infectious bursal disease virus vaccine. Vet. Immunol. Immunopathol..

[17] Boo S.Y., Tan S.W., Alitheen N.B., Ho C.L., Omar A.R., Yeap S.K. (2020). Transcriptome analysis of chicken intraepithelial lymphocyte natural killer cells infected with very virulent infectious bursal disease virus. Sci. Rep..

[18] Eldaghayes I., Rothwell L., Williams A., Withers D., Balu S., Davison F., Kaiser P. (2006). Infectious bursal disease virus: Strains that differ in virulence differentially modulate the innate immune response to infection in the chicken bursa. Viral Immunol..

[19] Huang X., Liu W., Zhang J., Liu Z., Wang M., Wang L., Zhou H., Jiang Y., Cui W., Qiao X. (2021). Very virulent infectious bursal disease virus-induced immune injury is involved in inflammation, apoptosis, and inflammatory cytokines imbalance in the bursa of fabricius. Dev. Comp. Immunol..

[20] Xu Z.-Y., Yu Y., Liu Y., Ou C.-B., Zhang Y.-H., Liu T.-Y., Wang Q.-X., Ma J.-Y. (2019). Differential expression of pro-inflammatory and anti-inflammatory genes of layer chicken bursa after experimental infection with infectious bursal disease virus. Poult. Sci..

[21] Abou El-Fetouh M.S., Hafez M.H., El-Attar E.-S.R., El-Agamy M.E., Ali A. (2021). Comparative bursal cytokine gene expression and apoptosis in vaccinated chickens following virulent infectious bursal disease virus challenge. Virology.

[22] Ali B.H. (2010). Comparative immunologic and physiologic study of Broiler vaccinated with five different Gumboro vaccines: Balqees H. Ali, Emad J. Khammas. Iraqi J. Vet. Med..

[23] Ameen S.M., Adel A., Selim A., Magouz A., AboElKhair M., Bazid A.H. (2022). A multiplex real-time reverse transcription polymerase chain reaction assay for differentiation of classical and variant II strains of avian infectious bronchitis virus. Arch. Virol..

[24] Aricibasi M., Jung A., Heller E.D., Rautenschlein S. (2010). Differences in genetic background influence the induction of innate and acquired immune responses in chickens depending on the virulence of the infecting infectious bursal disease virus (IBDV) strain. Vet. Immunol. Immunopathol..

[25] Livak K.J., Schmittgen T.D. (2001). Analysis of relative gene expression data using real-time quantitative PCR and the 2^−ΔΔCT^ method. Methods.

[26] Hao X., Li S., Li J., Yang Y., Qin A., Shang S. (2021). An Anti-Tumor Vaccine Against Marek’s Disease Virus Induces Differential Activation and Memory Response of γδ T Cells and CD8 T Cells in Chickens. Front. Immunol..

[27] Liu H., Zhang M., Han H., Yuan J., Li Z. (2010). Comparison of the expression of cytokine genes in the bursal tissues of the chickens following challenge with infectious bursal disease viruses of varying virulence. Virol. J..

[28] Adu-Asiamah P., Zhang Y., Amoah K., Leng Q.Y., Zheng J.H., Yang H., Zhang W.L., Zhang L. (2021). Evaluation of physiological and molecular responses to acute heat stress in two chicken breeds. Animal.

[29] Bancroft J.D., Layton C. (2012). The hematoxylins and eosin. Bancroft’s Theory Pract. Histol. Tech..

[30] Teshome M., Fentahunand T., Admassu B. (2015). Infectious bursal disease (*Gumboro disease*) in Chickens. Br. J. Poult. Sci..

[31] Rashid M., Luo H., Akhter J., Islam M., Islam M., Rahman M., Cao Y., Xue C. (2013). Protection effect of Vaxxitek HVT+ IBD vaccine against infectious bursal disease in broiler chickens. Progress. Agric..

[32] Zhuo Z., Chen M., Zhou Z., Chen M. (1998). Discussion on the causes for the outbreaks of IBD in immunized chicken flocks. Chin. J. Vet. Med..

[33] Le Gros F., Dancer A., Giacomini C., Pizzoni L., Bublot M., Graziani M., Prandini F. (2009). Field efficacy trial of a novel HVT-IBD vector vaccine for 1-day-old broilers. Vaccine.

[34] Alam J., Rahman M., Sil B., Khan M. (2002). Giasuddin, and MSK Sarker. Effect of maternally derived antibody on vaccination against infectious bursal disease (Gumboro) with live vaccine in broiler. Int. J. Poult. Sci..

[35] Knoblich H., Sommer S., Jackwood D. (2000). Antibody titers to infectious bursal disease virus in broiler chicks after vaccination at one day of age with infectious bursal disease virus and Marek’s disease virus. Avian Dis..

[36] Goddard R., Wyeth P., Varney W. (1994). Vaccination of commercial layer chicks against infectious bursal disease with maternally derived antibodies. Vet. Rec..

[37] Zorman Rojs O., Krapež U., Slavec B., Juršič-Cizerl R., Poljanec T. (2011). Field efficacy of different vaccines against infectious bursal disease in broiler flocks. Acta Vet. Hung..

[38] Chansiripornchai N., Sasipreeyajan J. (2009). Comparison of the efficacy of the immune complex and conventionally live vaccine in broilers against infectious bursal disease infection. Thai J. Vet. Med..

[39] Chang H.-C., Lin T.-L., Wu C.-C. (2003). DNA vaccination with plasmids containing various fragments of large segment genome of infectious bursal disease virus. Vaccine.

[40] Chang H.C., Lin T.L., Wu C.C. (2001). DNA-mediated vaccination against infectious bursal disease in chickens. Vaccine.

[41] Lemiere S., Gauthier J.-C., Kodjo A., Vinit L., Delvecchio A., Prandini F. (2013). Evaluation of the Protection against Infectious Bursal Disease (IBD) Challenge in Progeny Born to Parents Having Received a Vaccination Program Using a Herpesvirus of Turkey-Infectious Bursal Disease (HVT-IBD) Vector Vaccine. World J. Vaccines.

[42] Bose R.K., Hossain K.M., Sil B.K., Taimur M., Pugliese C., Franci O. (2003). Comparative sero evaluation of live and killed Gumboro vaccine in broilers. Ital. J. Anim. Sci..

[43] Schijns V.E., van de Zande S., Lupiani B., Reddy S.M. (2014). Practical aspects of poultry vaccination. Avian Immunology.

[44] Wyeth P., Chettle N. (1990). Use of infectious bursal disease vaccines in chicks with maternally derived antibodies. Vet. Rec..

[45] Zahid B., Aslam A., Qazi J., Ahmad N., Ara C., Akhtar R., Bacha U. (2017). Pathogenicity and immunosuppressive effect of different vaccines of Infectious Bursal Disease virus. JAPS J. Anim. Plant Sci..

[46] Tsukamoto K., Saito S., Saeki S., Sato T., Tanimura N., Isobe T., Mase M., Imada T., Yuasa N., Yamaguchi S. (2002). Complete, long-lasting protection against lethal infectious bursal disease virus challenge by a single vaccination with an avian herpesvirus vector expressing VP2 antigens. J. Virol..

[47] Tsukamoto K., Kojima C., Komori Y., Tanimura N., Mase M., Yamaguchi S. (1999). Protection of chickens against very virulent infectious bursal disease virus (IBDV) and Marek’s disease virus (MDV) with a recombinant MDV expressing IBDV VP2. Virology.

[48] Tsukamoto K., Sato T., Saito S., Tanimura N., Hamazaki N., Mase M., Yamaguchi S. (2000). Dual-viral vector approach induced strong and long-lasting protective immunity against very virulent infectious bursal disease virus. Virology.

[49] Rautenschlein S., Yeh H., Sharma J. (2003). Comparative immunopathogenesis of mild, intermediate, and virulent strains of classic infectious bursal disease virus. Avian Dis..

[50] Jin X., Ogg G., Bonhoeffer S., Safrit J., Vesanen M., Bauer D., Chen D., Cao Y., Demoitie M.-A., Zhang L. (2000). An antigenic threshold for maintaining human immunodeficiency virus type 1-specific cytotoxic T lymphocytes. Mol. Med..

[51] Rauf A., Khatri M., Murgia M.V., Saif Y.M. (2011). Expression of perforin–granzyme pathway genes in the bursa of infectious bursal disease virus-infected chickens. Dev. Comp. Immunol..

[52] Tanimura N., Sharma J. (1997). Appearance of T cells in the bursa of Fabricius and cecal tonsils during the acute phase of infectious bursal disease virus infection in chickens. Avian Dis..

[53] Kaiser P., Rothwell L., Galyov E.E., Barrow P.A., Burnside J., Wigley P. (2000). Differential cytokine expression in avian cells in response to invasion by Salmonella typhimurium, Salmonella enteritidis and Salmonella gallinarum. Microbiology.

[54] Kim I.-J., Karaca K., Pertile T., Erickson S., Sharma J. (1998). Enhanced expression of cytokine genes in spleen macrophages during acute infection with infectious bursal disease virus in chickens. Vet. Immunol. Immunopathol..

[55] Boyman O., Sprent J. (2012). The role of interleukin-2 during homeostasis and activation of the immune system. Nat. Rev. Immunol..

[56] Jang D.-i., Lee A.-H., Shin H.-Y., Song H.-R., Park J.-H., Kang T.-B., Lee S.-R., Yang S.-H. (2021). The role of tumor necrosis factor alpha (TNF-α) in autoimmune disease and current TNF-α inhibitors in therapeutics. Int. J. Mol. Sci..

[57] Hersperger A.R., Pereyra F., Nason M., Demers K., Sheth P., Shin L.Y., Kovacs C.M., Rodriguez B., Sieg S.F., Teixeira-Johnson L. (2010). Perforin expression directly ex vivo by HIV-specific CD8+ T-cells is a correlate of HIV elite control. PLoS Pathog..

[58] Kuerten S., Nowacki T.M., Kleen T.O., Asaad R.J., Lehmann P.V., Tary-Lehmann M. (2008). Dissociated production of perforin, granzyme B, and IFN-γ by HIV-specific CD8+ cells in HIV infection. AIDS Res. Hum. Retroviruses.

[59] Campbell T., Coles E. (1986). Avian clinical pathology. Vet. Clin. Pathol..

[60] Sultan H., Hussein H.A., Abd El-Razik A.G., El-Balall S., Talaat S.M., Shehata A.A. (2012). Efficacy of HVT-IBDV vector vaccine against recent Egyptian vvIBDV in commercial broiler chickens. Int. J. Poult. Sci..

[61] Kumar K., Singh K., Prasad C. (2000). Immune responses to intermediate strain IBD vaccine at different levels of maternal antibody in broiler chickens. Trop. Anim. Health Prod..

