# Correction: Gewaily et al. Cytokines, Serological, and Histopathological Assessment of Recombinant Vaccination Strategies for Combatting Infectious Bursal Disease in Broiler Chickens. *Vaccines* 2024, *12*, 27

**DOI:** 10.3390/vaccines13090914

**Published:** 2025-08-28

**Authors:** Mahmoud S. Gewaily, Fares El-Khyat, Abd Elnaby Tahoon, Mohammed Al-Rasheed, Safaa E. Abdo, Ahmed Gado, Mohamed Elmasry, Mahmoud M. Ismail

**Affiliations:** 1 Department of Anatomy and Embryology, Faculty of Veterinary Medicine, Kafrelsheikh University, Kafrelsheikh 33516, Egypt; 2 Department of Poultry and Rabbit Diseases, Faculty of Veterinary Medicine, Kafrelsheikh University, Kafrelsheikh 33516, Egyptmahmoud.ismail@vet.kfs.edu.eg (M.M.I.); 3 Animal Health Research Institute, Kafrelsheikh Branch, Kafrelsheikh 33511, Egypt; 4 Department Clinical Sciences, College of Veterinary Medicine, Avian Research Center, King Faisal University, P.O. Box 400, Al-Ahsa 31982, Saudi Arabia; 5 Department of Animal Wealth Development, Faculty of Veterinary Medicine, Kafrelsheikh University, Kafrelsheikh 33516, Egypt; 6 Department of Poultry and Fish Diseases, Faculty of Veterinary Medicine, Damanhour University, Damanhour 22511, Egypt; 7 Agricultural Research Center, Animal Production Research Institute, Animal Production Research Station, Sakha, Kafrelsheikh 33511, Egypt

The authors would like to make the following corrections to this published paper [1]:

In the original publication, there was an unintentional misarrangement of the primers during the formatting of Table 1 before submission. Specifically, the primers (listed in the second left column) were inadvertently shifted relative to their corresponding gene names. This error occurred during the population of the table and does not reflect inaccuracies in the actual experimental procedures or primer design.

The corrected Table 1 appears below.

In the original publication, Figure S1 (Assessment of RNA integrity, purity, and melting curves for some investigated cytokine genes) was correctly presented; however, there were missing letters (D, E, F) in the caption on the supplementary Figure S1 that referred to the melting curves. The sentence in line 3

“C; Representative melting curves for some investigated genes, including GAPDH gene, Perforin, interferon (INF), and IL2.”

should change to

“[C-F]: Representative melting curves for some investigated genes, including GAPDH, Perforin, IFN-γ, and IL2, respectively.”

The authors state that the scientific conclusions are unaffected. This correction was approved by the Academic Editor. The original publication has also been updated.

## Figures and Tables

**Table 1:** Primers of investigated genes with annealing temperature, accession number, and reference for each gene.

Gene	Primer Sequence	Annealing Tm	Accession NO/Reference
IFN-γ	F: CTCCCGATGAACGACTTGAG R: CTGAGACTGGCTCCTTTTCC	60	NM_205149.2 [26]
TNF-α	F: CGCTCAGAACGACGTCAA R: GTCGTCCACACCAACGAG	60	MF000729.1 [26]
Granzyme A	F: CCTGATACTTCCTGGAGATTTGTGC R: TTTTTGTCCGTGAATGGGCTC	60	NM_204457.1 [26]
Perforin	F: CACCCGCACCAAAAGATGAAG R: CGCCTCCTGGAAAACACACAAC	60	KC551799.1 [26]
Il 2	F: TTCTGGGACCACTGTATGCTCTT R: TACCGACAAAGTGAGAATCAATCAG	60	AF000631.1 [27]
IL10	F: CAGACCAGCACCAGTCATCA R: TCCCGTTCTCATCCATCTTCTC	62	NM_001004414.2 [28]
IL1B	F: TGCCTGCAGAAGAAGCCTCG R: GACGGGCTCAAAAACCTCCT	62	NM_204524.1 [28]
GAPDH	F: TGCCATCACAGCCACACAGAAG R: ACTTTCCCCACAGCCTTAGCAG	60	AF047874.1 [27]
