# Supplementary material for: Cytokines, Serological, and Histopathological Assessment of Recombinant Vaccination Strategies for Combatting Infectious Bursal Disease in Broiler Chickens
Source: Vaccines (Basel). 2023 Dec 26;12(1):27. doi: 10.3390/vaccines12010027 (PMC10818727; doi:10.3390/vaccines12010027)
Supplement: Supplementary file 1 [file vaccines-12-00027-s001.zip › vaccines-2756860-Supplementary.pdf]

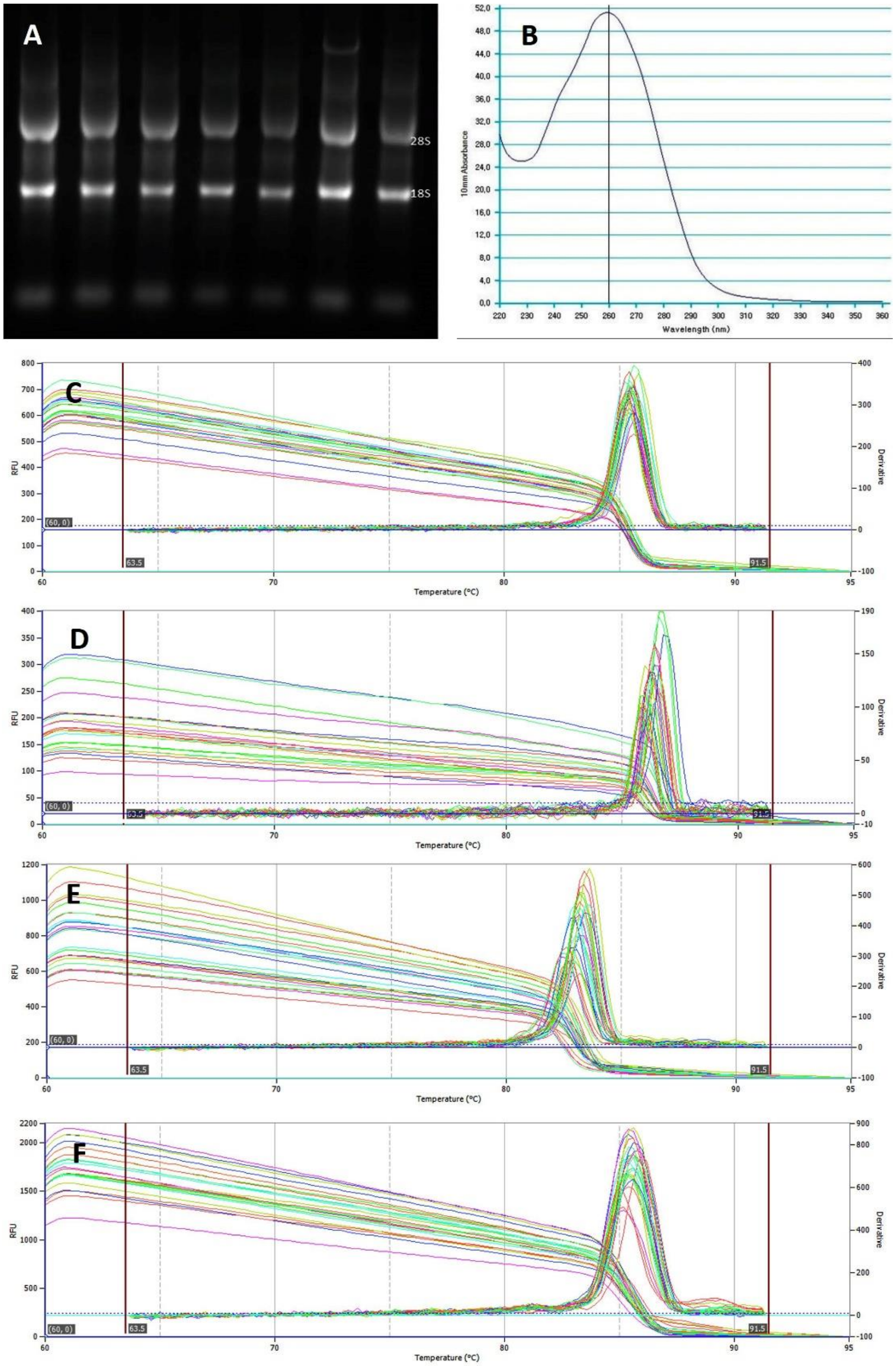

Figure S1. The RNA integrity was assessed using gel electrophoresis (2%) which displayed intact 28S and 18S rRNA (A) in 7 representative samples. The purity was assessed by Nanodrop and determination of 260/280 ratios (B). C; Representative melting curves for some investigated genes including GAPDH gene, Perforin, interferon (INF), and IL2. For all the tested samples, the dissociation-curve analysis showed only one peak at about (84-86°C).
